# Developing integrated community-based HIV prevention, harm reduction, and sexual and reproductive health services for women who inject drugs

**DOI:** 10.1186/s12978-019-0711-z

**Published:** 2019-05-29

**Authors:** Sylvia Ayon, Fatma Jeneby, Faizah Hamid, Abdalla Badhrus, Taib Abdulrahman, Gitau Mburu

**Affiliations:** 1 Kenya AIDS NGO Consortium, Nairobi, Kenya; 2 Muslim Education and Welfare Association, Mombasa, Kenya; 3 REACHOUT Trust, Mombasa, Kenya; 4 Division of Health Research, University of Lancaster, Lancaster, LA1 4YW UK; 5 Department of Reproductive Health and Research, World Health Organization, Geneva, Switzerland

**Keywords:** HIV, Reproductive health, Contraception, Harm reduction, Heroin, Integration, Gender, Kenya

## Abstract

**Background:**

Despite being a priority population for HIV prevention and harm reduction programs, the sexual and reproductive health (SRH) needs of women who inject drugs are being overlooked. Furthermore, models for providing integrated SRH, HIV, and harm reduction services for women who inject drugs are rare. This article reports the development of community-based outreach services that integrated family planning and other SRH interventions with HIV and harm reduction services for this population in coastal Kenya.

**Methods:**

Using mixed-methods implementation research, a qualitative baseline needs assessment was conducted with women who inject drugs and harm reduction stakeholders using a combination of in-depth interviews and focus group discussions. The qualitative data from participants was subjected to thematic analysis using Nvivo. Based on the baseline needs assessment, integration of SRH into existing HIV and harm reduction services was implemented. After two years of implementation, an evaluation of the program was conducted using a combination of qualitative interviews and review of quantitative service delivery records and other program documents. The process, impacts, and challenges of integrating SRH into a community-based HIV prevention and harm reduction program were identified.

**Results:**

This article highlights: 1) low baseline utilization of family planning services among women who inject drugs, 2) improved utilization and high acceptability of outreach-based provision of SRH services including contraception among this population, 3) importance of training, capacity strengthening, technical support and financial resourcing of community-based organizations to integrate SRH into HIV prevention and harm reduction services, and 4) the value of beneficiary involvement, advocacy, and collaboration with other partners in the planning, designing and implementing of SRH interventions for women who inject drugs.

**Conclusions:**

Women who inject drugs in this study had low utilization of family planning and other SRH services, which can be improved through the integration of contraceptive and other SRH interventions into existing outreach-based HIV prevention and harm reduction programs. This integration is acceptable to women who inject drugs, and is programmatically feasible. For successful integration, a rights-based beneficiary involvement, coupled with sustainable technical and financial capacity strengthening at the community level is essential.

**Electronic supplementary material:**

The online version of this article (10.1186/s12978-019-0711-z) contains supplementary material, which is available to authorized users.

## Background

Injecting drug use is an emerging driver of the Human Immunodeficiency Virus (HIV) epidemic in Kenya [1–3]. Recent data shows that 18.7% of injecting drugs are infected with HIV nationally, which is over three times the national prevalence of 5.6% [4, 5]. In this context, heroin is the most injected drug, with 93% of injecting drug users reporting using it in a recent epidemiological study [6]. In response to the growing burden of HIV among people who inject drugs in Kenya, the Ministry of Health endorsed a harm reduction approach in the national HIV strategy in 2013 [7], and in the following year, introduced harm reduction interventions for opioid dependence [8], primarily consisting of needle/syringe exchange programs, medically assisted therapy with methadone [9], and HIV testing and treatment for people who inject drugs.

Despite efforts to scale up harm reduction interventions for people who inject drugs in Kenya, a large number of women are unreached with these services [10, 11], yet females comprise over a tenth of the 18, 000 people who inject drug nationally [4, 12]. Partly due to their limited access to harm reduction services, Kenyan women who inject drugs are particularly affected by HIV, with its prevalence reaching 20% among those at the coast [5, 6]. As such, focusing on this population is essential in mitigating HIV and other harms of injecting drug use.

The vulnerability of women who inject drugs not unique to Kenya. In many parts of the world, women who inject drugs face severe gender, social and economic inequalities that adversely affect their health and well-being. In many countries, women who inject drugs are likely to have multiple sexual partners [13, 14] and to engage in sex work to support their drug use [14], which increases their vulnerability to HIV infection. Despite the majority of the 3.5 million women who inject drugs globally being of reproductive age [15, 16], their gender-specific needs are largely overlooked [17–19]. These women have limited access to drug treatment [20] and other essential services such an antenatal care globally [21, 22]. In countries such as Kenya where contraceptive prevalence is 47% [23], they may have an even higher need for family planning services [24]. In the Kenyan context, a failure to meet contraception needs of women who inject drugs could also accelerate transmission of HIV to their children, given the high prevalence of HIV in this population.

Given the above concerns, it is imperative to ensure that women who inject drugs in Kenya have access to a comprehensive package of gender-sensitive sexual and reproductive health (SRH) services tailored to their needs and circumstances. However, there is a shortage of documented models for providing family planning and other SRH interventions for women who inject drugs in Kenya [11], where harm reduction services are still nascent [25]. This article documents lessons learnt from the integration of SRH services into a community-based outreach program, so as to inform potential replication elsewhere.

### Community-based outreach programs

Although the response to injecting drug use has traditionally been based around residential -based rehabilitation, there is an emerging view that community-based treatment should be offered as an alternative to incarceration whenever possible [26]. In contrast to residential-based drug treatment, community-based treatment is primarily reliant on neighborhood outreach approach. Community-based outreach programs ascribe to certain key principles, including: minimal disruption to existing support systems, comprehensive continuum of care, evidence-based practices, acceptance of services, and cultural appropriateness [26].

As opposed to relying on people who inject drugs to attend health facilities, community-based outreach programs utilise outreach workers to reach out to drug users in their own localities, providing them with clean needles/syringes, HIV testing and educational messages [27]. Typically, community-based organizations (CBOs) provide services through outreach and static drop-in centers. Through this model, community outreach workers are the frontline peer educators, who are in constant contact with people who inject drugs [28]. Apart from providing HIV testing, needles and syringes during outreach, outreach workers also encourage people who inject drugs to regularly access other services from linked drop-in centers. Outreach workers often include people who formerly injected drugs [29]. Such a peer-led approach enables people who understand issues faced by people who inject drugs to actively participate in the provision of harm reduction services. By implementing harm reduction services through local CBOs and drop-in centers, the approach utilizes existing community infrastructure. A key feature of CBOs and drop-in centers is their closeness, familiarity and acceptability to people who injecting drugs.

## Methods

### Setting

In the two Kenyan coastal towns of Mombasa and Kilifi, community-based harm reduction services were implemented through a partnership between KANCO, a local non-governmental organization, and two implementing local CBOs, namely REACH OUT and MEWA.

### Implementation research problem

During implementation, routine program review showed that fewer women were accessing needles, syringes, HIV testing, and other harm reduction services. Furthermore, SRH services such as pregnancy testing and contraception are essential, yet these were generally not included within existing interventions. The lack of family planning interventions within harm reduction programs was not necessarily unique, as this has also been observed in other settings [21, 30, 31]. However, given the high rates of HIV prevalence in coastal Kenya, provision of contraception and other SRH services was deemed essential as it could have additional benefits of preventing mother to child transmission of HIV.

To facilitate the development of a community-based program that better responded to the needs of women who inject drugs, the program set forth a process of SRH needs assessment, gathering perspectives from women who inject drugs, as well as stakeholders who were closely involved in service provision to people who inject drugs in the study setting. The development of integrated services was designed as action research in which beneficiaries, harm reduction staff and external stakeholders were involved in identifying the research questions, implementing solutions, and identifying lessons learnt to continually improve the overall services and practices within the program.

In the formative phase, the project team sought to answer the following research questions:

1. What are the specific SRH service needs of women who inject drugs in Mombasa and Kilifi, Kenya?
2. What are the social determinants of access to sexual reproductive health services among women who inject drugs in Mombasa and Kilifi?
3. What factors hinder access to sexual and reproductive health services among women who inject drugs in Mombasa and Kilifi?

In an evaluative phase, which occurred after an initial 2-year implementation of integrated services, the following questions were addressed:

4) What is the outcome of capacity building activities on SRH integration to CBOs?
5) What is the impact of integrating SRH into community-based outreach services for women who inject drugs?
6) What challenges remain to integrating SRH into community-based outreach services for women who inject drugs?

### Research design

The study used mixed-method approach, utilizing multiple sources of data. Primary sources of data were qualitative in-depth interviews (IDIs) and focused group discussions (FGDs) with women who inject drugs, as well as key stakeholders who had an interest in or were involved in providing services to these women. These qualitative data sources were complemented with secondary data from programmatic reports and service delivery records at the two CBOs. Such mixed-method approaches are widely used to link or contextualize quantitative and qualitative data within implementation research [32, 33].

### Data collection

Data were collected in two phases: a formative phase to inform program design focusing on research questions 1–3 above, and an evaluative phase focusing on research questions 4–6 above.

#### Formative phase interviews and focus group discussions

To understand perspectives regarding their access to SRH services, in-depth interviews and FGDs were held with 45 women who inject drugs. Participants were invited to take part in the study by outreach workers during outreach, or at drop-in centres. Those who accepted were scheduled for appointment. To be included, women had to be over 18 years of age to allow independent consent, be within the reproductive age bracket of 18–49 years, and have injected drugs in the last 90 days. Of the 45 women, 24 participated in interviews (12 in each site) and another 21 participated in three FGDs (2 sessions in Mombasa and 1 session in Kilifi). Interviews and FGDs covered current drug use, contraceptive use, pregnancy experiences, and HIV testing. Apart from the women, in-depth interviews were conducted with 5 key stakeholders. These stakeholder interviews aimed to triangulate women’s perspectives as recommended by other scholars [34], and to obtain contextual information to aid in the design of the program. The key stakeholders interviewed in the formative phase included a community health worker (*n* = 1), outreach workers (*n* = 2), a Ministry of Health official (*n* = 1), and an outreach manager (*n* = 1). All in-depth interviews and FGDs were conducted in Swahili or English, were audio recorded, and lasted between 45 and 60 min.

#### Evaluative phase interviews

The second phase of data collection aimed to elicit initial reactions to the expanded services that included SRH interventions. Similar to the formative phase, two groups of participants were interviewed: women who inject drugs and key stakeholders. From among the women who inject drugs, in-depth interviews were conducted with 14 representatives. Subsequently, 30 key stakeholders were interviewed, who included program managers and technical officers (*n* = 25), and outreach workers (*n* = 5). In both phases of data collection, key stakeholders were sampled purposively in consultation with representatives from the two CBOs. Selection of key stakeholders was based on their interest, role in service provision to women who inject drugs, or policy expertise related to either injecting drug use or SRH. The evaluative-phase interviews also focused on the impact and remaining challenges of integrating SRH services into the program, were conducted in Swahili or English, were audio recorded, and lasted an average of 45 min.

#### Retrospective analysis of program records

The above qualitative data were supplemented with program reports that included service delivery records and training reports. Data related to 1) trainings, 2) SRH interventions in the 12 months prior to integration (baseline), and 3) 24 months of integrated SRH service implementation were extracted from training reports and service registers of the two CBOs, as appropriate.

### Data analysis

In-depth interviews and FGDs were transcribed and translated into English as appropriate and analyzed using Nvivo (QSR International), which is a useful software for conducting computer-assisted analysis of qualitative data [35]. This qualitative analysis was guided the overarching action research questions to draw out emerging themes. In this article, we focus on findings related to family planning and SRH contexts of participants. In addition to the thematic analysis, data relating to program activities was summarized using Microsoft Excel, and is used in this article to contextualize qualitative findings.

### Ethical considerations

Data were collected in private rooms. Consent was obtained from each participant after a detailed description of the study objective and procedures was provided to them. All participants were informed that they retained a right to withdraw at any time during the in-depth interviews or FGDs. Ethical review and approval of this study was provided by the National Commission for Science Technology and Innovation (ref: P/15/8861/4510).

## Results

### Needs assessment results

#### Contraceptive use among women who injected drugs

The average participant had at least 1 child. Of the 45 women, 37 women had at least one child (range 1–5). Overall, 29% were using contraceptives at the time of the study, while over two thirds (69%; *n* = 31) were not. Among women who were using contraceptives, most (13%; *n* = 6) were relying on condoms for contraception and protection from HIV. Very few participants reported using long-acting methods such as injections (7%), or implants (4%), and none reported using intra-uterine contraceptive devices (Table 1).

#### Women’s perception of their own family planning needs

Accounts from women suggested that their contraceptive use was low. In a typical response, when asked about present or past use of contraceptive, one participant stated that “*I have never used any family planning method*” (Participant # 9, Mombasa), while another responded jovially as follows: “*Haha, I don’t have. I don’t have any method of contraception*” (Participant # 9, Kilifi). However, women were cognizant of the need to plan for children:

*Life is hard, you can’t get pregnant and be able to take care of the child when you are still a drug user.* (Participant # 5, Kilifi)

Several women reported that transport costs were to blame for their low contraceptive use. One participant explained that “*getting time to go to other hospitals and use money to receive family planning services*” prevented her from using contraceptives (Participant # 9, Mombasa). In other cases, women reported that they were not using contraception because “*I don’t have the information about those things*” (Participant # 6, Mombasa). In addition, virtually all the women involved in this study experienced amenorrhea due to drug use, and this reduced their perceived need for family planning. One women who was not using contraception claimed that her drug use had “*become my way of family planning*”, and further elaborated by stating that:

*Given the way in which I am using drugs, I don’t think that I will get pregnant any time soon.* (Participant # 3, Kilifi)

In several cases, side effects were blamed for the low utilisation of  contraceptives. For example, one participant reported that she “*developed side effects like difficulty in breathing, and adding weight, then I decided to leave them alone*” (Participant # 9, Kilifi).

#### Stakeholders perspectives of women’s contraception and SRH needs

Key stakeholders who were directly involved in the provision of services indicated the lack of SRH services, including family planning commodities:

*People who using drugs should be offered family planning. Reproductive health is a right for everybody, but for women who inject drugs, it is more serious because they frequently get unwanted pregnancies. They should be using family planning and condoms –male and female condoms –, but most of them don’t have access to them*. (Key stakeholder # 1, Community Health Worker, Kilifi)

During in-depth interviews, several stakeholders expressed high levels of interest to integrate family planning and other SRH services into the community-based outreach services, while emphasizing that SRH needs of women had been a neglected issue:

*The issue of reproductive health has been a challenge. The level of women’s access to these services is very low, but you know: people who use drugs have been neglected for long and it has now become a big issue. We have had families in the dens and rehabs, children are growing up in the dens, and access to pre-natal and post-natal care is low. Women should be a priority in this; we need programs supporting and addressing reproductive issues of women who inject drugs.* (Key stakeholder # 1, Outreach Worker, Mombasa)

These findings from women and stakeholders – which identified potential demand and barriers to access to SRH services – were essential in informing the design, planning and development of a new set of SRH interventions that could be integrated into the community-based outreach services. At the same time, it was essential to explore the resource requirement and other contextual considerations that should be taken into account in integrating SRH interventions into the program.

#### Resource needs

Several stakeholders highlighted a lack of resources as a key barrier to the provision of family planning and other SRH services for women who inject drugs. CBO managers reported a lack of budget to hire qualified nurses with SRH expertise to implement women-specific interventions. The lack of funds was particularly notable among the CBOs as they depended on external fund-raising to implement services. Stakeholders emphasized that while they were aware that a need to provide SRH services existed, “t*he challenge we have is that if we want have these services provided we have to set aside some money for them*”. (Key stakeholder # 1, Community Health Worker, Kilifi).

#### Considerations in the design of the intervention

Several other issues came to the fore regarding the implementation of the project including stigma, safety and security, meaningful engagement of beneficiaries, and the need to bring the services closer to women. In addition, stakeholders from CBOs indicated that women who were arrested or imprisoned did not have access to SRH services while in custody. On their part, women reported willingness to access SRH services at the CBOs, and identified outreach and other staff at CBOs as key sources of information and services. Asked for her opinion about how family planning and other SRH services could be ideally provided to her and her peers, a participant who was at a drop-in center said that “*first of all, they need to bring the service here because many women are lazy to go there [health facility]; sometimes they lack fare. It [family planning service] should be brought here to this drop-in center”.* (Participant # 1, Kilifi).

Stakeholders reported that the surrounding communities had been opposed to free distribution of needles and syringes to people who inject drugs. In this context, drug use was still stigmatized, especially among women. Communities were reported to ascribe to the view that that “*it is a shame for a woman to be a drug user*” (Stakeholder # 3, Program Manager, Kilifi). As such, abstinence rather than harm reduction was the goal prescribed by community values. These findings highlighted a need for advocacy to transform community norms and perception regarding harm reduction services for people who inject drugs, as well as ensuring that safety and security was maintained. Together the above findings were subsequently taken into consideration when integrating family planning and other SRH interventions into the existing outreach services.

### Design and implementation of the intervention

#### Training

Between 2014 and 2015, KANCO provided competency-based training on the provision of gender-sensitive services to women who inject drugs to CBO staff. A total of 51 staff were trained (16 in 2014 and 35 in 2015) from the two CBOs. The training focused on SRH needs of women who inject drugs as well as female partners of men who inject drugs. Topics covered included behavior change communication, contraception, cervical cancer screening, prevention, testing and treatment of HIV, screening, diagnosis and treatment of sexually transmitted infections (STIs), partner notification and referrals, ante-natal and post-natal care, and two-way referrals for a range of other services. CBO staff were coached on how to take relevant medical and drug use details from people who use drugs, and how to adopt and maintain friendly attitudes towards them at all times. All trainings emphasized the importance of rights-based approaches to services as well as the surveillance, documentation, and response to gender-based violence.

#### Organizational, financial and technical support

Over the same period, KANCO provided organizational support to the two CBOs to strengthen their financial and program management procedures, and provided sub-grants to the two CBOs to directly finance SRH activities. Additional technical support was provided to enhance tailoring of outreach and drop-in modes of services to meet the needs of women, including procurement and distribution of menstrual hygiene packages and diapers for women with infants. The CBOs were linked to the national reproductive health commodity management system for quantification, ordering and management of other SRH commodities such as condoms and oral contraceptive pills. Technical guidelines, toolkits, reference manuals, and resources related to provision of comprehensive services for people who inject drugs were printed and distributed at the CBOs, and training conducted on novel concepts or recommendations. At the central level, KANCO staff participated in the harm reduction sub-committee of the Key Population Technical Working Group, which is responsible for development of guidelines, tools, and research agenda related to key populations nationally. Their participation in the Technical Working Group facilitated downstream cascading of new technical resources to CBOs.

#### Strengthening human resource capacity

A nurse with reproductive health training was appointed to specifically lead the SRH component at each CBO. The reproductive health nurse was an addition to the existing multi-disciplinary team at each CBO that comprised of a CBO manager, outreach coordinator and supervisor, clinical officer, community-outreach workers/ peer educators, and monitoring and evaluation/data officers. Rarely, paralegals were involved to support victims of gender-based violence.

#### Integration of family planning services into community-based outreach services

Once the requisite resources and technical capacity was in place, the CBOs expanded their core HIV and harm reduction services to include family planning and other SRH interventions. The expanded repertoire of SRH services included onsite pregnancy testing, short-term contraceptives, and cervical cancer screening. In addition, referrals for more complicated services to nearby government and non-governmental health facilities were intensified. Outreach workers and peer educators mobilized women who inject drugs at drug dens, surrounding neighborhoods, temporary shelters, and their homes. Outreach staff from the CBOs reached out to the target population, provided the above SRH services, and referred clients to drop-in centers and to other government or non-governmental health facilities based on their needs. The referral network established during this project involved a range of partners such as International Center for Reproductive Health, the AIDS Healthcare Foundation, Marie Stopes International, Family Health Options Kenya, Bomu Hospital, Coast Provincial General Hospital, Malindi District Hospital, and two HIV comprehensive care clinics at the latter two hospitals. The full complement of services provided through this program is shown in the following Table 2.

**Table 1:** Fertility and contraception use among the study sample at baseline

Characteristic	IDI	FGDs	Total	%
Number of children (mean)	1.4	1.8	1.6	–
Current contraceptive				
Condoms	5	1	6	13%
Calendar	1	0	1	2%
Implant	2	0	2	4%
Herbal	1	0	1	2%
None	13	18	31	69%
Injection	1	2	3	7%
Unknown	1	0	1	2%

**Table 2:** Expanded services provided to women who inject drugs at the study sites

Service domain	Interventions and services provided during outreach	Interventions and services provided at drop-in centres	Referrals to private and government health and social services
Prevention and treatment of HIV and co-infections.	Condoms, HIV testing, information, communication and education on HIV and sexually transmitted infections.	HIV testing and counselling.	Referral for confirmation and treatment of HIV, and screening of Hepatitis C and Tuberculosis.
Harm reduction.	Clean needle and syringes, alcohol swabs, cotton wool.	Addiction counseling and first aid for violence and overdose.	Referrals for medically assisted therapy with methadone.
Sexual and reproductive health services.	Information on family planning, sister-to-sister counselling, hygiene packages/tampons and oral contraceptive pills.	Pre-natal education, and provision of short-acting reversible contraceptives.	Referral for long-acting long-acting reversible contraception, ante-natal care, and screening for cervical cancer.
Personal, social and child care services.	Transportation to health facilities and provision of personal care items.	Personal care (shower, toothbrush/paste, lotion), short-term shelter, and diapers for children.	Referrals for sexual and physical gender-based violence (GBV) and legal assistance.

#### Safety, security and advocacy as part of program implementation

Given the initial resistance of the local community towards provision of needles and syringes to people who inject drugs, the implementing partners reinforced collaborative working with local gate keepers and communities to ensure that the overall goal and purpose harm reduction was understood. Initially, local communities were against provision of clean needles and syringes and other harm reduction interventions which they thought would encourage injecting drug use, and several trainings and workshops were interrupted by community members. To mitigate such risks, an assessment of security and safety was included as part of initial capacity building and service provision activities.

In addition, the project team held a series of public community meetings with the police, members of parliament, administrative chiefs, women groups, and other community members to disseminate factual information about harm reduction approach. Regular communication and security meetings chaired by provincial commissioner of police and community members were conducted. As part of the project advocacy strategy, several community representatives, CBO representatives, Ministry of Health employees, and county government administrative officials were taken on an exposure visit to Mauritius to witness the operations and impact of an established harm reduction program that included SRH services. These representatives later became ardent supporters of the program and allayed the concerns of the surrounding communities. Additionally, a number of initial outreaches were ‘blended’ whereby government SRH officers accompanied outreach teams to provide services to women who inject drugs. This strategy aimed to mitigate drug-use stigma by demonstrating to the public that outreach activities were supported by the Ministry of Health.

#### Blended outreach to prisons

Based on the needs assessments, blended outreach was conducted in women’s prisons, such as Shimo-la-Tewa Women Prison, to reach incarcerated women. These outreach services included activities and services targeted at promoting the SRH for women at the prisons.

#### Engagement of people who inject drugs in service planning, monitoring and advocacy

Given assertions from stakeholders that needs of people who inject drugs were perennially ignored, the program created opportunities for women who inject drugs to participate in district health committee meetings, which were the main forums where issues, needs and concerns related to local health service availability and provision were discussed. Participation of people who inject drugs in these forums allowed them to advocate for their needs and provide feedback information to/from their peers regarding current and planned services.

### Impact of integrating SRH services into community-based outreach program

#### Increase in provision of SRH services

**Fig. 1:**
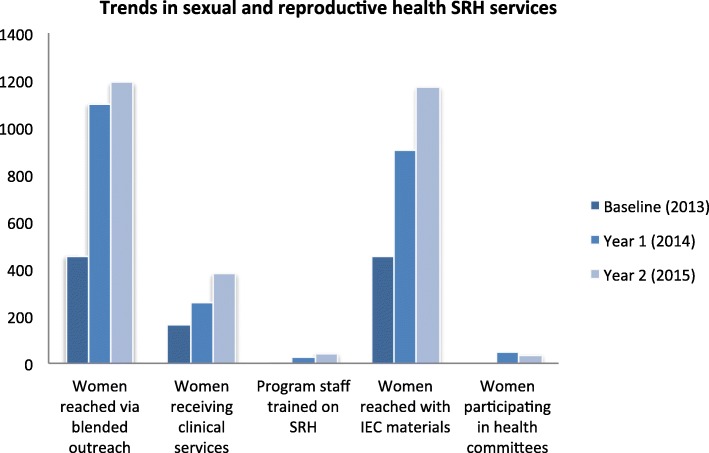
Trends in SRH services provided to women who inject drugs at two CBOs

Over the two-year period there was notable increase in the number of women reached with relevant interventions. From negligible numbers in 2013, a total of 2262 women were reached through outreach, 626 were provided with clinical services, 2096 were provided with educational materials, and 92 participated in district health committees between 2014 and 2015 (Fig. 1).

Of the women who received clinical services 88% received HIV testing, almost all received condoms, a third (31%) received pregnancy testing, a third (31%) received short term combined oral contraceptives, 3.5% received long-term contraceptives on site (at one CBO) or through referrals, 29% were treated for STIs, 24% were screened for cervical cancer, and 1% received post-GBV emergency contraception and counselling (which were all the women who reported sexual assaults). Apart from providing services during outreach and at drop-in centers, more than 330 women were transported or bi-directionally referred to nearby government or non-governmental health facilities for long-term contraceptive insertion, post-abortion care, antiretroviral therapy, and other advanced clinical services.

#### Perceptions regarding integrated services

Data suggested that integration of family planning and other SRH services into the outreach program attracted women to harm reduction services in general, including to drop-in centers:

*I have seen change as an outreach worker. I have seen them improve in terms of their interest in needles and syringes because of this sexual and reproductive element.* (Key Stakeholder # 2, Kilifi)

Stakeholders reported that women showed consistent interest in the integrated services that included SRH components. For example, a CBO program manager asserted that “*we have seen that clients themselves are very committed; they want these services*” (Key Stakeholder # 3, Kilifi). This interest was particularly notable given that prior to the inclusion of SRH services, provision harm reduction outreach services was not necessarily tailored to the needs of women who inject drugs:

*We didn’t have a specific package for females, and there was no project that was addressing issues of women. So we started implementing this innovative SRH project, which has brought great mileage.* (Key Stakeholder # 1, Mombasa)

In contrast to the previous situation, integration of SRH into the harm reduction outreach services was said to be “*bringing about positive change”* as it was *“benefitting a lot of women who have SRH needs that had remained unaddressed for a long time”* (Key Stakeholder # 1, Mombasa).

#### Perceptions regarding trainings and technical support

Findings from CBOS staff suggested that the approach of linking capacity building activities to needs assessment was particularly valuable in responding to the most pertinent barriers of CBO-based provision of SRH services:

*Training enabled us to expand services to include family planning. It also gave us ideas on how to attract women who inject drugs, which was initially a challenge*. (Key Stakeholder # 3, Kilifi)

In relation to continuity of SRH services to the women, another stakeholder asserted  that “*before getting the training, it was difficult for us to get women to access SRH services and to link them to the referral clinics, but after the training, we have been able to link and follow them up*” (Key Stakeholder # 1, Program Manager, Mombasa).

## Discussion

Within the global response to the needs of people who inject drugs, the contraceptive and broader SRH needs of women who inject drugs are needs are easily overlooked [19] due to a systematic failure to integrate gender-sensitive interventions into harm reduction services [17, 36]. In the midst of limited models for providing integrated SRH, HIV and harm reduction services, this article describes the process, initial outcomes and challenges of integrating SRH services into community-based outreach services for women who inject drugs in coastal Kenya. Four key findings warrant discussion.

First is that the utilization of contraceptives among women who inject drugs was generally low. Evidence from other parts of the world show that women who inject or use drugs are less likely to use reliable and effective contraception [37–39], and are more likely to have unplanned pregnancies, compared with non-drug-using women [40]. In a country such as Kenya where contraception prevalence rate is low at 47% [23], understanding contraception and fertility-related behaviors of marginalised women who inject drugs is critical in ensuring that their needs are met and universal access to sexual and reproductive health and rights is realised for all. In particular, our study demonstrates the utility of participatory action research in identifying needs and desired models of providing integrated services to stigmatized women who inject drugs.

Second, this study shows that community-based outreach is a feasible model through which family planning and other SRH services can be provided. Qualitative findings suggested that this model was acceptable to women who inject drugs, and was preferable compared to other forms of facility based provision that forced women to incur transport costs. Evidence from other countries suggest that merely referring women who inject drugs to external facilities to access SRH services is often not effective in meeting their needs because most referrals are not completed [31]. In the study context, majority of services were provided at the community level, resorting to external referrals only for advanced services, and these were generally accompanied through transportation, or tracked through two-way referral slips as previously reported [11]. At the same time, ensuring that services were provided in spaces that women were familiar and comfortable with was particularly important to the success of the integration, and the peer-led outreach model and drop-in centers provided that environment.

Third, an observation that drug-induced amenorrhea was common and it prevented women from perceiving the need for family planning was made in our study. We have highlighted this issue in a separate publication [24], but note that it is not unique to our sample, as it has been observed in other countries [30, 41]. However, in our study, women were reached with interpersonal messages emphasizing that they could still get pregnant despite this phenomenon, which, combined with service provision at outreach and drop-in centers ensured that family planning messages and services were incorporated at each HIV and harm reduction service point.

Fourth, our study highlights the value of training, human resource capacity building, technical support and financial resourcing of CBOs to provide contraception and other SRH services. Trainings relating to how to provide gender-sensitive services to women, combined with strengthening of program management tools, dissemination of technical resources, and the provision of financial sub-grants to CBOs, all facilitated the expansion of SRH services tailored to women who inject drugs. Although these are common avenues of capacity building [42], they were particularly important given a previous  observation that a lack of capacity is a common barrier to service delivery by small CBOs in Kenya [43]. By strengthening CBO systems and increasing their access to finances through sub-granting, the two CBOs in this project were able to enhance their human resources and technical capacity required for service provision tailored to women who inject drugs. In addition, imparting health providers with necessary skills and positive attitudes through trainings was relevant given that attitudes of health providers are known to affect women’s utilization of SRH services [44, 45].

### Limitations

This article provides preliminary findings of integration of family planning and SRH services into a community-based drug treatment program using a small sample of participants. Future studies may wish to explore this on a larger scale. This study’s findings may be limited in its generalizability beyond the study context: it included women who were in contact with an outreach-based HIV prevention and harm reduction services, who many differ from those without such contact. It is also possible that the findings reported here may have been affected by social response bias as noted in other studies of people who inject drugs [46]. These limitations notwithstanding, this article presents useful information and lessons learnt in designing and integrating family planning and wider SRH services into community-based harm reduction services for women who inject drugs, which can inform replication in other settings.

## Conclusion

Women who inject drugs tend to have low utilization of family planning and other SRH services. At the same time, findings from this study suggest that integrating SRH interventions into community-based outreach services for women who inject drugs is feasible and acceptable to women who inject drugs, and has the potential to increase women’s uptake of contraception, to curb HIV infections, and could benefit their children by preventing vertical transmission of HIV. To successfully integrate SRH into community-based harm reduction programs, it is essential to strengthen organizational and human resource capacity, technical support, and financial resources of CBOs, and community acceptability of integrated programs through advocacy.

A French translation of this article has been included as Additional file 1.

A Portuguese translation of the abstract has been included as Additional file 2.

## Additional files

Additional file 1: Translation of this article into French. (PDF 325 kb)

Additional file 2: Translation of the abstract of this article into Portuguese. (PDF 96.7 kb)

## Abbreviations

CBO: Community-based organization
FGD: Focus group discussion
HIV: Human Immunodeficiency Virus
IDI: In-depth interview
KANCO: Kenya AIDS NGO Consortium
MEWA: Muslim Education and Welfare Association
NGO: Non-governmental organization
SRH: Sexual and Reproductive Health
