# Supplementary material for: Developing integrated community-based HIV prevention, harm reduction, and sexual and reproductive health services for women who inject drugs
Source: Reprod Health. 2019 May 29;16(Suppl 1):59. doi: 10.1186/s12978-019-0711-z (PMC6538559; doi:10.1186/s12978-019-0711-z)
Supplement: Supplementary file 1 — Translation of this article into French. (PDF 325 kb) [file 12978_2019_711_MOESM1_ESM.pdf]

Développement de services communautaires intégrés de prévention du VIH, réduction des méfaits et santé sexuelle et reproductive pour les femmes faisant usage de drogues injectables.

Sylvia Ayon[1], Fatma Jeneby[2], Faizah Hamid[3], Abdalla Badhrus[2], Taib Abdulrahman[3], Gitau Mburu[4*]

1. Kenya AIDS NGO Consortium (Consortium des ONG SIDA au Kenya), Nairobi, Kenya.
2. Muslim Education and Welfare Association (Association musulmane d'éducation et d'aide sociale), Mombasa, Kenya.
3. Reach Out Centre Trust (Centres de proximité pour le traitement et la prévention), Mombasa, Kenya.
4. Département de recherche médicale de l'université de Lancaster, Lancaster, Royaume-Uni.

Adresses électroniques :
SA: sayon@kanco.org
FJ: fjeneby@yahoo.com
BH: bintyhamid@gmail.com
AB: abdallabadhrus@yahoo.com
TA: twayeebahmed@gmail.com
GM: g.mburu@lancs.ac.uk

*Auteur correspondant : Gitau Mburu, Division of Health Research, University of Lancaster, Lancaster, LA1 4YW, United Kingdom. Email: g.mburu@lancs.ac.uk

Résumé

Contexte : Bien que les femmes faisant usage de drogues injectables constituent une population prioritaire pour les programmes de prévention du VIH et de réduction des méfaits, leurs besoins en matière de santé sexuelle et reproductive (SSR) sont négligés. Par ailleurs, les modèles proposant des services intégrés de SSR, prévention du VIH et réduction des méfaits pour les femmes faisant usage de drogues injectables sont rares. Cet article présente le développement de services communautaires de proximité intégrant la planification familiale et d'autres actions SSR aux services de prévention du VIH et de réduction des méfaits pour cette population sur la côte kenyane.

Méthodes : S'appuyant sur la recherche-action, une évaluation qualitative des besoins de base a été menée auprès de femmes faisant usage de drogues injectables et de personnes concernées par la réduction des méfaits. Cette évaluation a pris la forme d'entretiens approfondis (EA) et de groupes de discussions thématiques (GDT). Les données qualitatives recueillies chez les participants ont été soumises à une analyse thématique avec Nvivo. Suite à l'évaluation des besoins de base, l'intégration des services de SSR aux services de prévention du VIH et de réduction des méfaits a été mise en oeuvre. Deux ans après sa mise en place, le programme a été évalué. Pour ce faire, des entretiens qualitatifs ont été menés

et des rapports quantitatifs sur les prestations de services et d'autres documents liés au programme ont été consultés. Le processus, les impacts et les défis liés à l'intégration de services de SSR dans un programme communautaire de prévention du VIH et de réduction des méfaits ont été également identifiés.

Résultats : Cet article souligne : 1) un recours peu fréquent aux services de planification familiale chez les femmes faisant usage de drogues injectables, 2) une meilleure utilisation et un niveau d'acceptabilité élevé des services SSR de proximité, notamment pour la contraception, dans cette population, 3) l'importance de la formation, du renforcement des capacités, du support technique et des ressources financières des organisations communautaires pour intégrer la SSR aux services de prévention du VIH et de réduction des méfaits et 4) la valeur apportée par l'implication des bénéficiaires, la sensibilisation et les partenariats constitués pour la planification, la conception et la mise en oeuvre des actions SSR pour les femmes faisant usage de drogues injectables.

Conclusions : Dans cette étude, les femmes faisant usage de drogues injectables avaient peu recours aux services de planification familiale et à d'autres services SSR. Il est possible d'améliorer cette situation en intégrant les actions relatives à la contraception et à la SSR dans des programmes de prévention du VIH et de réduction des méfaits déjà en place. Cette intégration est acceptable pour les femmes faisant usage de drogues et sa mise en place est réalisable. Cependant, une implication des bénéficiaires fondée sur les droits, ainsi qu'un renforcement durable des moyens techniques et financiers au niveau de la communauté, sont essentiels pour la réussite de cette intégration.

Mots clés : VIH, santé reproductive, contraception, réduction des méfaits, héroïne, intégration, sexe, Kenya.

Contexte

L'utilisation de drogues injectables est un moteur émergent de l'épidémie du virus de l'immunodéficience humaine (VIH) au Kenya [1-3]. Des données récentes montrent que 18,7 % des usagers de drogues injectables sont infectés par le VIH au niveau national, soit

trois fois plus que la prévalence nationale de 5,6 % [4, 5]. Dans ce contexte, l'héroïne est la drogue la plus consommée. En effet, selon une étude épidémiologique récente [6], 93 % des usagers de drogues injectables consomment de l'héroïne. En réponse au poids croissant du VIH pesant sur les usagers de drogues injectables au Kenya, le ministre kenyan de la Santé a approuvé en 2013 une approche de réduction des méfaits dans la stratégie nationale de lutte contre le VIH [7] et, l'année suivante, a mis en place des actions de réduction des méfaits pour le traitement de la dépendance aux opioïdes [8]. Ces actions prennent principalement la forme de programmes d'échange d'aiguilles et de seringues, de traitements médicament assistés à base de méthadone [9] et de tests de dépistage et traitements du VIH pour les usagers de drogues injectables.

En dépit des efforts réalisés pour étendre les actions de réduction des méfaits chez les usagers de drogues injectables au Kenya, un grand nombre de femmes n'ont pas accès à ces services [10, 11]. Pourtant, les femmes représentent plus d'un dixième des 18 000 usagers de drogues injectables dans le pays [4, 12]. En partie en raison de leur accès limité aux services de réduction des méfaits, les femmes kenyanes faisant usage de drogues injectables sont particulièrement touchées par le VIH, sa prévalence atteignant 20 % chez celles habitant sur la côte [5, 6]. Il est donc essentiel de se concentrer sur cette population pour réduire la propagation du VIH et les autres méfaits de l'utilisation de drogues injectables.

La vulnérabilité des femmes faisant usage de drogues injectables n'est pas propre au Kenya. Dans beaucoup d'endroits dans le monde, les femmes faisant usage de drogues injectables sont confrontées à des inégalités sociales, économiques et de genre qui affectent de manière négative leur santé et leur bien-être. Par ailleurs, dans de nombreux pays, les femmes faisant usage de drogues injectables sont susceptibles d'avoir plusieurs

partenaires sexuels [13, 14] et de se livrer à la prostitution pour financer leur consommation de drogues [14], ce qui augmente encore leur vulnérabilité face à l'infection par le VIH. Bien que la majorité des 3,5 millions de femmes faisant usage de drogues injectables soient en âge de procréer [15, 16], les besoins spécifiques à leur sexe sont largement négligés [17-19]. Ces femmes du monde entier ont un accès limité aux traitements médicamenteux [20] et à d'autres services essentiels comme les soins prénatals [21, 22]. Dans des pays comme le Kenya, où la prévalence contraceptive est de 47 % [23], elles ont peut-être même encore plus besoin de services de planification familiale [24]. En effet, étant donné la prévalence élevée du VIH chez les femmes faisant usage de drogues injectables au Kenya, une réponse insuffisante à leurs besoins de contraception pourrait accélérer la transmission du VIH de ces femmes à leurs enfants.

Étant donné les enjeux décrits ci-dessus, il est impératif de s'assurer que les femmes faisant usage de drogues injectables au Kenya aient accès à un ensemble complet de services de santé sexuelle et reproductive (SSR) spécifiques aux femmes et adaptés à leurs besoins et à leurs situations. Cependant, il y a une pénurie de modèles documentés de prestations de services de planification familiale et autres actions SSR destinées aux femmes faisant usage de drogues injectables au Kenya [11], pays dans lequel les services de réduction des méfaits sont toujours en cours de développement [25]. Cet article présente les leçons tirées de l'intégration de services SSR dans un programme communautaire de proximité afin de préparer d'éventuelles initiatives similaires ailleurs.

Programmes communautaires de proximité

Bien que la réponse à l'usage de drogues injectables soit généralement une admission dans un centre de désintoxication, on commence à se rendre compte que l'on

devrait proposer un traitement au sein de la communauté comme alternative à l'enfermement lorsque c'est possible [26]. Contrairement aux traitements de la toxicomanie pratiqués dans les centres de désintoxication, le traitement au sein de la communauté repose principalement sur la proximité des services. Les programmes de traitement communautaires s'appuient sur quelques principes clés, notamment : perturbation minimale des systèmes de soutien déjà en place, gamme complète de soins, pratiques éprouvées, acceptation des services et pertinence culturelle des soins [26].

Au lieu d'attendre que les usagers de drogues injectables se rendent dans des établissements de santé, les responsables des programmes communautaires de proximité font appel à du personnel de proximité qui va à leur rencontre, sur leur lieu de vie, et leur fournit des aiguilles/seringues propres, leur propose des tests de dépistage du VIH et leur délivre des messages pédagogiques [27]. Généralement, les organisations communautaires (CBO) combinent les services apportés sur le terrain et ceux proposés dans des centres d'accueil fixes. Dans ce modèle, le personnel de proximité de la communauté constitue une équipe d'éducateurs de première ligne, qui sont en contact permanent avec les usagers de drogues injectables [28]. Par ailleurs, les membres du personnel de proximité ne se contentent pas de proposer des tests de dépistage du VIH et de fournir des aiguilles et des seringues lorsque qu'ils vont sur le terrain, mais ils encouragent également les usagers de drogues injectables à se rendre régulièrement dans des centres d'accueil situés à proximité afin de profiter d'autres services. Les membres du personnel de proximité sont souvent d'anciens usagers de drogues injectables [29]. Cette approche centrée sur l'éducation par les pairs permet aux gens qui comprennent les problèmes rencontrés par les usagers de drogues injectables de participer activement aux prestations de services destinées à la réduction des méfaits. En mettant en place des services de réduction des méfaits par le biais

d'organisations communautaires (CBO) et de centres d'accueil locaux, cette initiative s'appuie sur les infrastructures communautaires existantes. L'un des éléments clés de ces CBO et de ces centres d'accueil est leur proximité, leur convivialité et leur acceptabilité par les usagers de drogues injectables.

Méthodes

Configuration

Dans les deux villes côtières kenyanes Mombasa et Kilifi, des services communautaires de réduction des méfaits ont été mis en place via un partenariat entre KANCO, une organisation non gouvernementale locale, et deux CBO locales, REACH OUT et MEWA.

Problèmes révélés par la recherche

Pendant la mise en place, l'évaluation des programmes courants a montré que peu de femmes avaient accès à des aiguilles, des seringues, des tests de dépistage du VIH ou d'autres services de réduction des méfaits. Par ailleurs, les services SSR, tels que les tests de grossesse et la contraception, sont essentiels, et pourtant ils n'étaient généralement pas inclus dans les initiatives existantes. Le manque de services de planification familiale dans les programmes de réduction des méfaits n'était cependant pas nécessairement unique, car ce phénomène a également été observé dans d'autres lieux [21, 30, 31]. Cependant, étant donné les taux élevés de prévalence du VIH dans la région côtière kenyane, il est apparu essentiel de proposer des méthodes de contraception et d'autres services SSR dans la mesure où cela permettrait également de réduire la transmission du VIH de la mère à l'enfant.

Pour faciliter le développement d'un programme communautaire plus adapté aux

besoins des femmes faisant usage de drogues injectables, une évaluation des besoins SSR a été proposée, l'objectif étant de recueillir l'option des femmes faisant usage de drogues injectables et celle des personnes étroitement impliquées dans les prestations de services apportées aux usagers de drogues injectables sur le lieu géographique concerné par l'étude. Ce développement de services intégrés a été conçu sous forme de recherche-action dans laquelle les bénéficiaires et les personnes impliquées dans la réduction des méfaits devaient aider à identifier les questions à se poser, mettre en place des solutions et tirer des conclusions afin d'améliorer continuellement les services et les pratiques du programme.

Pendant la phase d'élaboration, l'équipe de projet cherchait à répondre aux questions suivantes :

1) Quels sont les besoins SSR spécifiques des femmes faisant usage de drogues injectables à Mombasa et Kilifi, au Kenya ?
2) Quels sont les déterminants sociaux d'accès aux services de santé sexuelle et reproductive chez les femmes faisant usage de drogues injectables à Mombasa et Kilifi ?
3) Quels sont les facteurs qui freinent l'accès aux services de santé sexuelle et reproductive chez les femmes faisant usage de drogues injectables à Mombasa et Kilifi ?

Pendant la phase d'évaluation, qui a eu lieu après une période initiale de mise en place des services intégrés de 2 ans, les questions suivantes ont été abordées :

4) Quel est l'impact des activités de renforcement des capacités sur l'intégration des services SSR dans les CBO ?

5) Quel est l'impact de l'intégration des services SSR aux services communautaires de proximité pour les femmes faisant usage de drogues injectables ?
6) Quels sont les derniers défis à relever pour intégrer les services SSR aux services communautaires de proximité pour les femmes faisant usage de drogues injectables ?

Conception de l'étude

L'étude reposait sur une méthode mixte, avec plusieurs sources de données. Les sources de données primaires étaient des entretiens approfondis et des groupes de discussions thématiques avec des femmes faisant usage de drogues injectables et des personnes intéressées ou impliquées dans les services apportés à ces femmes. Ces sources de données qualitatives étaient complétées par des données secondaires issues de rapports sur les prestations de services et les programmes existants dans les deux CBO. Les méthodes mixtes de ce type sont largement utilisées pour lier ou contextualiser les données quantitatives et qualitatives dans une recherche sur la mise en oeuvre d'un projet [32, 33].

Collecte des données

Les données ont été collectées en deux phases : une phase d'élaboration visant à préparer la conception du programme , reposant sur les questions 1 à 3 ci-dessus, et une phase d'évaluation, reposant principalement sur les questions 4 à 6 ci-dessus.

Entretiens et groupes de discussions thématiques au cours de la phase d'élaboration : Pour comprendre le point de vue des femmes faisant usage de drogues injectables en termes d'accès aux services SSR, des entretiens approfondis ont été menés et des groupes de

discussions thématiques ont été constitués avec 45 de ces femmes. Ce sont des membres du personnel de proximité qui ont invité ces femmes à participer à l'étude, en les rencontrant soit sur leur lieu de vie, soit dans des centres d'accueil. Un rendez-vous a été fixé avec celles ayant accepté l'invitation. Pour pouvoir participer, ces femmes devaient avoir plus de 18 ans pour pouvoir donner leur consentement indépendant, être en âge de procréer (dans la tranche d'âge comprise entre 18 et 49 ans) et avoir pris des drogues injectables au cours des 90 derniers jours. Sur les 45 femmes, 24 ont participé à des entretiens (12 dans chaque site) et 21 ont participé à 3 groupes de discussions thématiques (2 sessions à Mombasa et 1 session à Kilifi). Les entretiens et les groupes de discussions thématiques portaient sur leurs expériences en matière de consommation de drogues, d'utilisation de méthodes de contraception, de grossesse et de tests de dépistage du VIH. Des entretiens approfondis ont également été menés avec 5 parties prenantes clés. Ces entretiens approfondis visaient à cerner le point de vue des femmes, conformément aux recommandations faites par d'autres chercheurs [34], et à obtenir des informations contextuelles utiles pour la conception du programme. Les parties prenantes clés interrogées pendant la phase d'élaboration étaient des professionnels de santé (n=1), des membres du personnel de proximité (n=2), un représentant officiel du Ministère kenyan de la Santé (n=1) et un responsable des services de proximité (n=1). Tous les entretiens et groupes de discussions thématiques ont eu lieu en Swahili ou en anglais, ont fait l'objet d'un enregistrement audio et ont duré entre 45 et 60 minutes.

Entretiens au cours de la phase d'évaluation : La seconde phase de collecte des données visait à provoquer de premières réactions suite à la mise en place de services étendus incluant des actions SSR. Comme pour la phase d'élaboration, deux groupes de participants

ont été interrogés : les femmes faisant usage de drogues injectables et les principales parties prenantes. Des entretiens approfondis ont été menés avec 14 représentantes des femmes faisant usage de drogues injectables. Ensuite, 30 parties prenantes clés ont été interrogées, notamment des responsables de programmes et des agents techniques (n=25), ainsi que des membres du personnel de proximité (n=5). Dans les deux phases de collecte des données, des parties prenantes clés ont été sélectionnées intentionnellement, en consultation avec des représentants des deux CBO. Le choix des parties prenantes clés était basé sur leur intérêt, leur rôle dans les prestations de services proposées aux femmes faisant usage de drogues injectables ou leur expertise en matière de stratégies relatives à l'usage de drogues injectables ou de SSR. Les entretiens de la phase d'évaluation étaient centrés sur l'impact de l'intégration de services SSR au programme et sur les derniers défis à relever. Ils ont eu lieu en Swahili ou en anglais, ont fait l'objet d'un enregistrement audio et ont duré 45 minutes en moyenne.

Analyse rétrospective des rapports du programme : Les données qualitatives ci-dessus ont été complétées par des rapports du programme, notamment des rapports de prestations de services et des rapports de formation. Des données relatives aux 1) formations, 2) actions SSR effectuées dans les 12 mois précédant l'intégration (période de référence), et 3) 24 mois de mise en place de services SSR intégrés ont été extraites des rapports de formation et des registres de services, et certaines ont été fournies par les deux CBO, le cas échéant.

Analyse des données

Les entretiens approfondis et les discussions des groupes thématiques ont été transcrites et

traduites en anglais, le cas échéant, puis analysées avec Nvivo (QSR International), logiciel d'analyse qualitative de données [35]. L'analyse qualitative, orientée par les questions générales de la recherche-action, a mis en lumière des thèmes émergents. Dans cet article, nous nous concentrons sur les résultats relatifs à la situation des participants dans le domaine de la planification familiale et de la SSR. En plus de l'analyse thématique, les données relatives aux activités du programme ont été également résumées à l'aide de Microsoft Excel, et elles sont utilisée dans cet article pour contextualiser les résultats qualitatifs.

Considérations éthiques

Les données ont été collectées dans des salles privées. Chaque participant a reçu une description détaillée de l'objectif et des procédures de l'étude et a donné son consentement. Tous les participants ont été informés qu'ils avaient le droit de se retirer de l'étude à tout moment pendant les entretiens ou les séances des groupes de discussions thématiques. L'évaluation éthique et l'approbation de cette étude ont été obtenues de la National Commission for Science Technology and Innovation (Commission nationale kenyane pour les sciences, la technologie et l'innovation) (réf. : P/15/8861/4510).

Résultats

Résultats de l'évaluation des besoins

Utilisation actuelle de méthodes de contraception par les femmes faisant usage de drogues injectables : En moyenne, les participantes avaient au moins 1 enfant. Sur les 45 femmes, 37 femmes avaient au moins un enfant (âgé de 1 à 5 ans). Globalement, 29 % de ces femmes avaient recours à des méthodes de contraception au moment de l'étude,

alors que plus des deux tiers (69 %, n=31) n'en utilisaient pas. Parmi les femmes qui utilisaient des méthodes de contraception, la plupart (13 %, n=6) utilisaient des préservatifs comme méthode de contraception et comme moyen de protection contre le VIH. Très peu de participantes disaient utiliser des méthodes à action prolongée comme des injections (7 %) ou des implants (4 %), et aucune ne disait utiliser un dispositif intra-utérin (**Tableau 1)**.

Point de vue des femmes sur leurs propres besoins en matière de planification familiale

Les témoignages de ces femmes suggéraient qu'elles avaient peu recours aux méthodes de contraception. Parmi les réponses types lorsqu'on les interrogeait sur leur utilisation présente ou passée de méthodes de contraception, une participante a dit : *« Je n'ai jamais utilisé de méthode de planification familiale »* (Participante n° 9, Mombasa), tandis qu'une autre a répondu sur un ton enjoué : *« Ha, ha, je n'en ai pas. Je n'ai pas de méthode de contraception »* (Participante n° 9, Kilifi). Cependant, ces femmes étaient conscientes qu'elles avaient besoin de planifier la conception de leurs enfants :

> *La vie est dure, on ne peut pas se retrouver enceinte et s'occuper d'un enfant si on se drogue encore.* (Participante n° 5, Kilifi)

Plusieurs femmes ont indiqué que les coûts des transports étaient responsables de leur faible recours aux méthodes de contraception. Une participante a expliqué qu'elle ne pouvait pas employer de méthode de contraception car il fallait *« du temps pour se rendre dans d'autres hôpitaux et de l'argent pour accéder aux services de planification familiale »* (Participante n° 9, Mombasa). Dans d'autres cas, des femmes ont déclaré qu'elles n'avaient pas recours à la contraception pour la raison suivante : *« Je ne connais pas ces choses-là »*

(Participante n° 6, Mombasa). Par ailleurs, pratiquement toutes les femmes impliquées dans cette étude ont connu une aménorrhée due à l'usage de drogues et cela leur a donné l'impression qu'elles avaient moins besoin de recourir à la planification familiale. L'une de ces femmes qui n'utilisait pas de moyen de contraception a même affirmé que sa consommation de drogue était devenue *« ma méthode de planification familiale »*, et elle a même ajouté :

> *« Vu la consommation de drogues que je fais, je pense qu'il risque de se passer du temps avant que je me retrouve enceinte ».* (Participante n° 3, Kilifi)

Certaines femmes ont également expliqué qu'elles utilisaient peu les méthodes de contraception en raison de leurs effets secondaires. Par exemple, l'une des participantes a déclaré qu'elle avait *« développé des effets secondaires comme une difficulté à respirer ou une prise de poids, donc j'ai décidé d'arrêter »* (Participante n° 9, Kilifi).

***Point de vue des parties prenantes sur les besoins des femmes en matière de contraception et de SSR** :* Les principales parties prenantes qui étaient directement impliquées dans les prestations de services ont signalé un manque de services SSR, notamment de structures de planification familiale :

> *Il faudrait proposer des services de planification familiale aux personnes qui se droguent. La santé reproductive est un droit pour tout le monde, mais il est plus important pour les femmes faisant usage de drogues injectables car elles sont fréquemment exposées à des grossesses non désirées. Elles devraient avoir recours à des méthodes de planification familiale et utiliser des préservatifs, masculins et féminins, mais la plupart n'y ont pas accès*. (Partie prenante clé n° 1, professionnel de santé communautaire, Kilifi)

Pendant les entretiens approfondis, plusieurs parties prenantes ont exprimé un grand intérêt pour l'intégration de services de planification familiale et d'autres services SSR aux services communautaires de proximité, tout en soulignant que les besoins SSR des femmes avaient été négligés :

> *Le problème de la santé reproductive constitue un véritable défi. Le niveau d'accès des femmes à ces services est très faible. Par ailleurs, les usagers de drogues injectables sont négligés depuis longtemps et c'est maintenant devenu un gros problème. Certaines familles vivent dans des taudis ou se trouvent dans des centres de désintoxication. Les enfants grandissent dans les taudis et l'accès aux soins prénatals et post-natals est faible. Les femmes devraient être prioritaires. Il nous faut des programmes qui répondent aux questions de reproduction des femmes faisant usage de drogues injectables et les accompagnent.* (Partie prenante clé n° 1, membre du personnel de proximité, Mombasa)

Les informations collectées auprès des femmes et des parties prenantes, qui ont permis d'identifier les demandes potentielles et les freins à l'accès aux services SSR, étaient essentielles pour définir la conception, la planification et le développement d'un nouvel ensemble d'actions SSR qui pourraient être intégrées aux services communautaires de proximité. En parallèle, il était essentiel d'identifier les ressources nécessaires et les éléments contextuels à prendre en compte pour étendre le programme.

Ressources nécessaires : Plusieurs parties prenantes ont souligné que le manque de ressources était un frein important à la fourniture de services de planification familiale et d'autres services SSR à destination des femmes faisant usage de drogues injectables. Les

responsables des CBO ont signalé un manque de budget pour embaucher des infirmiers qualifiés et formés à la SSR pour mettre en place des actions spécifiques aux femmes. Ce manque de fonds était particulièrement remarquable dans les CBO car la mise en place des services qu'elles proposent dépend de financements externes. Les parties prenantes ont bien indiqué que, malgré leur connaissance de la nécessité de proposer des services SSR, *« le défi que nous devons relever est que, si nous voulons proposer ces services, nous devons mettre de l'argent de côté pour les financer »*. (Partie prenante clé n° 1, professionnel de santé communautaire, Kilifi)

Considérations à prendre en compte dans la conception de l'intervention : Plusieurs autres problèmes sont passés au premier plan concernant la mise en place du projet, notamment la stigmatisation, la sécurité et la sûreté, une véritable implication des bénéficiaires et la nécessité d'apporter les services au plus près des femmes. Par ailleurs, les parties prenantes des CBO ont indiqué que les femmes arrêtées ou incarcérées n'avaient pas accès aux services SSR pendent leur détention. De leur côté, les femmes ont indiqué qu'elles souhaitaient avoir accès à des services SSR dans les CBO, et elles ont identifié certains membres du personnel de proximité ou d'autres personnes travaillant dans les CBO comme leurs principaux fournisseurs d'informations et de services. Alors qu'on lui demandait son avis sur la planification familiale et les autres services SSR qui pourraient lui être proposés, à elle et à ses pairs, une participante qui se trouvait dans un centre d'accueil a dit : *« d'abord, il faut apporter les services ici parce que beaucoup de femmes ont la flemme d'aller là-bas [établissement de santé] ; certaines ne peuvent pas payer le transport. Ils [services de planification familiale] doivent être apportés ici, dans ce centre d'accueil ».* (Participante n° 1, Kilifi)

Les parties prenantes ont indiqué que les communautés environnantes étaient opposées à une distribution gratuite d'aiguilles et de seringues aux usagers de drogues injectables. Dans cet environnement, l'usage de drogues a toujours été stigmatisé, en particulier chez les femmes. Il a été signalé que les communautés portaient le regard suivant sur ce sujet : *« c'est une honte pour une femme de se droguer »* (Partie prenante n° 3, responsable du programme, Kilifi). Par conséquent, l'abstinence plutôt que la réduction des méfaits était l'objectif à atteindre selon les valeurs de la communauté. Ces informations ont mis en lumière l'importance de la sensibilisation pour transformer les normes de la communauté et la façon dont elle perçoit les services de réduction des méfaits pour les usagers de drogues injectables, ainsi que pour maintenir la sécurité et la sûreté. Les informations ci-dessus ont toutes été prises en compte pour envisager l'intégration des services de planification familiale et d'autres services SSR aux services de proximité existants.

Conception et mise en place des actions

Formation : Entre 2014 et 2015, KANCO a délivré au personnel des CBO des formations sur les compétences nécessaires pour proposer des services spécifiques aux femmes destinés aux femmes faisant usage de drogues injectables. Au total, 51 personnes ont été formées (16 en 2014 et 35 en 2018) dans les deux CBO. La formation était axée sur les besoins SSR des femmes faisant usage de drogues injectables et des partenaires féminines des hommes faisant usage de drogues injectables. Les sujets abordés étaient : la communication sur le changement de comportement, la contraception, le dépistage du cancer du col de l'utérus, la prévention, les tests de dépistage et le traitement du VIH, le dépistage, le diagnostic et le

traitement des infections sexuellement transmissibles, la transmission d'informations au partenaire et son orientation vers les services, les soins prénatals et post-natals, et une orientation dans les deux sens vers d'autres services. Le personnel des CBO a été formé sur la façon d'obtenir des informations pertinentes relatives à la santé et à la consommation de drogues auprès des usagers de drogues, ainsi que sur l'attitude amicale à adopter et à conserver en permanence avec ces personnes. Toutes les formations soulignaient l'importance d'une approche basée sur les droits aux services ainsi que de la surveillance de la violence à caractère sexiste, la collecte de preuves et la réponse à y apporter.

Soutien fonctionnel, financier et technique : Sur la même période, KANCO a apporté un soutien fonctionnel aux deux CBO afin de renforcer leurs procédures financières et de gestion des programmes et leur a fourni des subventions pour financer directement les activités SSR. Un soutien supplémentaire a également été apporté pour adapter davantage les services de proximité et des centres d'accueil aux besoins des femmes, notamment en leur distribuant des kits de protections hygiéniques et des couches pour celles qui avaient des enfants. Les CBO étaient en rapport avec le système national de gestion des structures de santé reproductive pour la quantification, les commandes et la gestion des autres fournitures SSR telles que les préservatifs et les pilules contraceptives. Des recommandations techniques, des outils, des manuels de référence et des supports liés à la fourniture de services complets aux usagers de drogues injectables ont été imprimés et distribués aux CBO, et des formations ont été dispensées sur les nouveaux concepts ou les nouvelles recommandations. Au niveau central, le personnel de KANCO a participé à un sous-comité sur la réduction des méfaits organisé par le groupe de travail technique national pour les populations clés, responsable du développement de recommandations,

d'outils et de programmes de recherche liés aux populations clés. Leur participation au groupe de travail technique a facilité la transmission des nouveaux supports techniques aux CBO.

Renforcement de la capacité des ressources humaines : Un membre du personnel infirmier formé à la santé reproductive a été nommé pour superviser spécifiquement la division SSR dans chaque CBO. Cette personne a été choisie pour rejoindre l'équipe multidisciplinaire existante présente dans chaque CBO, qui comprenait un responsable de la CBO, un coordinateur et superviseur de proximité, un responsable clinique, des membres du personnel communautaire de proximité / pairs éducateurs et des responsables de la surveillance et de l'évaluation / des données. Dans de rares cas, des techniciens juridiques ont été impliqués pour soutenir les victimes de violence à caractère sexiste.

Intégration de services de planification familiale aux services communautaires de proximité : Après la mise en place des ressources et des moyens techniques nécessaires, les CBO ont étendu leurs principaux services de réduction des méfaits et de prise en charge du VIH à des services de planification familiale et autres services SSR. L'extension de la gamme de services SSR comprenait des tests de grossesse sur site, des contraceptifs à court terme et un dépistage du cancer du col de l'utérus. Par ailleurs, les recommandations de se rendre dans des établissements de santé de proximité (gouvernementaux ou non) pour bénéficier de services plus complets ont été intensifiées. Les membres du personnel de proximité et les pairs éducateurs ont sensibilisé les femmes faisant usage de drogues injectables dans les lieux de consommation de drogues, dans le voisinage, dans les hébergements temporaires et chez elles. Le personnel de proximité des CBO s'est rendu auprès des populations ciblées,

leur a fourni certains des services SSR mentionnés ci-dessus et les a orientées vers des centres d'accueil et d'autres établissements de santé (gouvernementaux ou non) en fonction de leurs besoins. Le réseau d'établissements recommandés établi pendant ce projet comprenait un ensemble de partenaires tels que l'International Center for Reproductive Health (Centre international pour la santé reproductive), la fondation AIDS Healthcare Foundation, l'ONGI Marie Stopes International, l'ONG locale Family Health Options Kenya, l'hôpital Bomu Hospital, l'hôpital Coast Provincial General Hospital, l'hôpital Malindi District Hospital et deux départements cliniques proposant des soins complets pour le VIH au sein de ces deux derniers hôpitaux. L'ensemble des services complémentaires proposés dans ce programme est présenté dans le **Tableau 2** ci-dessous.

Sécurité, sûreté et sensibilisation dans le cadre de la mise en place du programme : Étant donné les réticences initiales de la communauté locale envers la distribution d'aiguilles et de seringues aux usagers de drogues injectables, les partenaires chargés de la mise en place du programme ont renforcé le travail de collaboration avec les gardiens des traditions et les communautés afin de s'assurer que l'objectif global de réduction des méfaits était bien compris. Au départ, les communautés étaient hostiles à la distribution d'aiguilles et de seringues propres et aux actions de réduction des méfaits car elles pensaient que cela encouragerait l'usage de drogues injectables, et elles avaient perturbé plusieurs ateliers et sessions de formation. Pour réduire ces risques, une évaluation de la sécurité a été incluse dans les activités initiales de renforcement des capacités et de prestations de services.

Par ailleurs, l'équipe du projet a organisé une série de réunions publiques communautaires avec la police, les membres du parlement, les responsables administratifs, les groupes de femmes et d'autres membres de la communauté afin de faire passer les

informations factuelles sur la stratégie de réduction des méfaits. Des réunions régulières sur la communication et la sécurité présidées par le commissaire de police local et les membres de la communauté ont également été organisées. Dans le cadre de la stratégie de sensibilisation du projet, plusieurs représentants de la communauté, des représentants des CBO, des employés du Ministère kenyan de la santé et des représentants administratifs officiels du gouvernement local ont été conviés à l'île Maurice pour une visite de présentation visant à démontrer l'utilité des opérations et l'impact d'un programme de réduction des méfaits bien établi comprenant des services SSR. Ces représentants sont ensuite devenus d'ardents défenseurs du programme et ont dissipé les craintes des communautés environnantes. Enfin, un nombre significatif d'initiatives de proximité initiales étaient « mixtes » dans la mesure où des responsable SSR du gouvernement accompagnaient les équipes de proximité qui proposaient des services aux femmes faisant usage de drogues injectables. Cette stratégie visait à atténuer la stigmatisation des usagers de drogues en démontrant au public que les activités de proximité étaient soutenues par le Ministère de la santé.

Un service de proximité mixte dans les prisons : S'appuyant sur l'évaluation des besoins, des initiatives de proximité mixtes ont été menées dans les prisons de femmes, telles que Shimo-la-Tewa Women Prison, afin d'atteindre les femmes incarcérées. Ces initiatives de proximité comprenaient des activités et des services tournés vers la promotion de la SSR pour les femmes en prison.

Implication des usagers de drogues injectables dans la planification des services, la surveillance et la sensibilisation : Suite aux affirmations des parties prenantes selon

lesquelles les besoins des usagers de drogues injectables étaient éternellement ignorés, le programme a donné la possibilité aux femmes faisant usage de drogues injectables de participer à des réunions du comité de santé local, principaux lieux de discussion des problèmes, besoins et craintes liés à la disponibilité et à la fourniture de services de santé locaux. La participation des usagers de drogues injectables à ces réunions leur a également permis d'exprimer leurs besoins et de transmettre à leurs pairs des informations sur les services existants et ceux prévus.

Impact de l'intégration de services SSR au programme communautaire de proximité :

Augmentation du nombre de prestations de services SSR réalisées : Sur cette période de deux ans, le nombre de femmes touchées a considérablement augmenté. Alors que les chiffres étaient négligeables en 2013, entre 2014 et 2015 : 2 262 femmes au total ont été touchées grâce à la stratégie de proximité, 626 ont bénéficié de services cliniques, 2 096 ont reçu des supports pédagogiques et 92 ont participé à des comités de santé locaux (**Figure 1**).

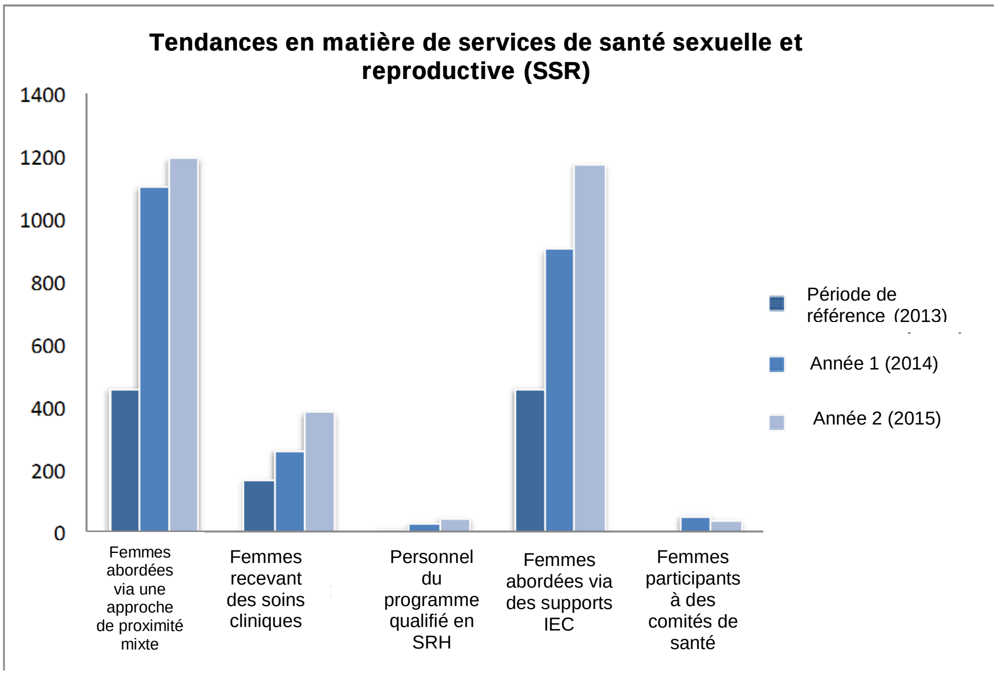

Figure 1. Tendances dans les services SSR fournis aux femmes faisant usage de drogues injectables dans deux CBO.

Parmi les femmes citées ci-dessus ayant bénéficié de services cliniques, 88 % ont fait un test de dépistage du VIH, pratiquement toutes ont eu des préservatifs, un tiers (31 %) ont reçu des tests de grossesse, un tiers (31 %) ont reçu des contraceptifs oraux combinés à court terme et 3,5 % ont reçu des contraceptifs à long terme sur site (dans une CBO) ou dans un endroit vers lequel elles ont été dirigées, 29 % ont été traitées pour des IST, 24 % ont été dépistées pour le cancer du col de l'utérus et 1 % ont reçu une contraception d'urgence post-GBV et des conseils (ce qui correspondait à toutes les femmes ayant signalé avoir subi des agressions sexuelles). Par ailleurs, en plus des prestations de services réalisées pendant les campagnes de proximité et dans les centres d'accueil, plus de 330 femmes ont été transportées ou orientées de manière bidirectionnelle vers des établissements de santé (gouvernementaux ou non) pour la pose d'un dispositif contraceptif à long terme, des soins post-avortement, un traitement antirétroviral et d'autres services médicaux avancés.

Perception des services intégrés : Les données suggèrent que l'intégration de services de planification familiale et d'autres services SSR au programme de proximité attirait les femmes vers les services de réduction des méfaits en général, notamment dans les centres d'accueil :

> *En tant que membre du personnel de proximité, j'ai vu la différence. J'ai vu leur intérêt augmenter pour les aiguilles et les seringues à cause de la dimension sexuelle et reproductive.* (Partie prenante clé n° 2, Kilifi)

Les parties prenantes ont déclaré que les femmes montraient un intérêt constant pour les services intégrés qui comprenaient des éléments SSR. Par exemple, un responsable de programme d'une CBO a affirmé que *« nous voyons que les bénéficiaires elles-mêmes sont très impliquées ; elles veulent ces services »* (Partie prenante clé n° 3, Kilifi). Cet intérêt est

particulièrement visible car, avant l'inclusion des services SSR, il était rare de fournir des services de proximité de réduction des méfaits aux femmes faisant usage de drogues injectables :

> *Nous n'avions pas de kit spécifique pour les femmes et il n'y avait pas de projet qui s'adressait aux problèmes des femmes. Alors nous avons commencé à mettre en place ce projet SSR innovant, et c'est vraiment payant.* (Partie prenante clé n° 1, Mombasa)

Contrairement à la situation précédente, l'intégration d'éléments SSR dans les services de proximité de réduction des méfaits a été jugée comme *« apportant un changement positif »* car elle *« bénéficiait à beaucoup de femmes dont les besoins SSR étaient négligés depuis longtemps »* (Partie prenante n° 1, Mombasa).

Perception des formations et du soutien technique : Les informations fournies par le personnel des CBO suggéraient que l'approche consistant à lier les activités de renforcement des capacités à l'évaluation des besoins était particulièrement bénéfique car elle s'attaquait à l'obstacle le plus pertinent concernant les prestations de services SSR proposées par les CBO :

> *Les formations nous ont permis d'étendre nos services pour inclure la planification familiale. Cela nous a également donné des idées sur la façon d'attirer les femmes faisant usage de drogues injectables, ce qui était au départ un véritable défi*. (Partie prenante clé n° 3, Kilifi)

Dans le cadre de la continuité des services SSR proposés aux femmes, une autre partie prenante a ajouté que *« avant la formation, il était difficile de les amener à accéder aux services et de les diriger vers les départements cliniques, mais après la formation, nous*

avons pu faire un suivi auprès d'elles » (Partie prenante n° 1, responsable de programme, Mombasa).

Discussion

Dans la réponse globale aux besoins des usagers de drogues injectables, la contraception et les besoins SSR plus larges des femmes faisant usage de drogues injectables sont facilement négligés [19] en raison d'un échec systématique à intégrer des actions spécifiques aux femmes dans les services de réduction des méfaits [17, 36]. Dans un milieu proposant peu de modèles de services intégrés de planification familiale, de traitement du VIH et de réduction des méfaits, cet article décrit le processus, les premiers résultats et les défis liés à l'intégration de services SSR aux services communautaires de proximité sur la côte kenyane. Quatre résultats clés méritent qu'on s'y attarde.

Premièrement, le recours à la planification familiale était généralement faible chez les femmes faisant usage de drogues injectables. Des faits avérés dans d'autres régions du monde montrent que les femmes faisant usage de drogues injectables ou consommatrices de drogues sont moins susceptibles d'avoir recours à une méthode de contraception fiable et effective [37-39] et risquent plus d'avoir des grossesses non désirées que les femmes ne faisant pas usage de drogues [40]. Dans un pays comme le Kenya, dans lequel le taux de prévalence contraceptive est faible (47 %) [23], la compréhension de l'usage de la contraception chez des femmes marginalisées faisant usage de drogues injectables et de leurs comportements par rapport à la fertilité est essentielle pour s'assurer que leurs besoins soient satisfaits et que l'accès universel à la santé et aux droits sexuels et reproductifs soit effectif pour tous. Notre étude démontre en particulier l'utilité d'une recherche-action participative dans l'identification des besoins et des modèles souhaités

pour les prestations de services destinées aux femmes stigmatisées faisant usage de drogues injectables.

Deuxièmement, cette étude montre que le système de proximité communautaire est un modèle envisageable pour des prestations de services de planification familiale et d'autres services SSR. Les résultats suggèrent que ce modèle était acceptable pour les femmes faisant usage de drogues injectables et qu'il était même préférable à d'autres formes de prestations dispensées dans des établissements éloignés, ce qui obligeait les femmes à payer des frais de transport. Des faits avérés dans d'autres pays suggèrent que si l'on se contente d'orienter des femmes faisant usage de drogues injectables vers des établissements externes pour bénéficier de services SSR, cela ne répond souvent pas à leurs besoins car la plupart du temps, elles ne s'y rendent pas [31]. Dans le contexte de l'étude, la plupart des services étaient fournis au niveau de la communauté, et les femmes étaient orientées vers des structures externes uniquement pour les services avancés. Elles étaient alors généralement accompagnées pendant le transport ou suivies grâce à un système de fiches dans les deux sens, comme cela a été indiqué précédemment [11]. Il était également particulièrement important pour la réussite de cette intégration de s'assurer que les services étaient fournis dans des lieux que les femmes connaissaient et où elles se sentaient bien. Le modèle de proximité géré par les pairs et les centres d'accueil correspondaient à cet environnement.

Troisièmement, l'observation révélant que lesaménorrhées liées à la consommation de drogue étaient fréquentes et empêchaient les femmes de se rendre compte de la nécessite de la planification familiale a été notée dans notre échantillon de l'étude. Nous avons souligné ce problème dans une publication séparée [24], mais il convient de noter qu'il n'est pas propre à notre échantillon. Il a en effet été observée dans d'autres pays [30,

41]. Cependant, dans notre étude actuelle, nous avons fait passer des messages interpersonnels aux femmes, leur expliquant qu'elles pouvaient tomber enceintes malgré ce phénomène. Cette initiative, associée aux prestations de services proposées sur le terrain et dans les centres d'accueil, a permis de s'assurer que les services et les messages relatifs à la planification familiale étaient intégrés à chaque point de service de traitement du VIH et de la réduction des méfaits.

Quatrièmement, notre étude souligne la valeur de la formation, du renforcement des capacités en ressources humaines, du soutien technique et des ressources financières destinés aux CBO pour les aider à proposer des services de contraception et d'autres services SSR. Les formations sur les prestations de services spécifiques aux femmes, les outils renforcés de gestion des programmes, la disponibilité des ressources techniques et l'octroi de subventions financières aux CBO ont facilité l'extension de services SSR adaptés aux femmes faisant usage de drogues. Bien que ces méthodes de renforcement de capacités soient courantes [42], elles étaient particulièrement importantes pour ces petites CBO kenyanes, qui souffrent très souvent d'un manque de moyens pour proposer leurs services [43]. Grâce à un renforcement de leurs systèmes et à une augmentation de leurs capacités financières via des subventions, les deux CBO impliquées dans ce projet ont pu améliorer leurs ressources humaines et leurs capacités techniques afin de proposer des services adaptés aux femmes faisant usage de drogues injectables. Les formations dispensées aux prestataires de soins pour leur transmettre les compétences requises et les attitudes positives à adopter ont été utiles car l'attitude des prestataires de soins est connue pour avoir un impact sur l'utilisation des services SSR par les femmes [44, 45].

Limites

Cet article fournit les premiers résultats de l'intégration des services de planification familiale et SSR au programme communautaire de traitement de la toxicomanie par le biais d'un petit échantillon de participants. Des études ultérieures seront menées pour poursuivre la recherche à plus grande échelle. La généralisation des résultats de cette étude peut être limitée au delà du contexte de l'étude. En effet, cette étude concernait des femmes qui étaient en contact avec des services de proximité de prévention du VIH et de réduction des méfaits, et beaucoup de ces femmes étaient différentes de celles qui n'ont pas ce contact. Il est également possible que les résultats signalés ici aient été affectés par des biais sociologiques dans les réponses, comme cela a été observé dans d'autres études sur les usagers de drogues injectables [46]. Malgré ces limites, cet article présente des informations utiles et des conclusions intéressantes sur la conception et l'intégration de services de planification familiale et d'autres services SSR plus larges aux services communautaires de proximité de réduction des méfaits pour les femmes faisant usage de drogues injectables, informations précieuses pour reproduire cette expérience dans d'autres environnements.

Conclusion

Les femmes faisant usage de drogues ont peu recours aux services de planification familiale et à d'autres services SSR. D'un autre côté, les résultats de cette étude laissent à penser que l'intégration d'actions SSR aux services communautaires de proximité pour les femmes faisant usage de drogues est réalisable et acceptable, et qu'elle a le potentiel d'augmenter le recours à une méthode de contraception pour ces femmes, de limiter les infections du VIH et qu'elle pourrait bénéficier à leurs enfants en empêchant la transmission verticale du VIH. Pour réussir l'intégration des services SSR aux programmes communautaires de

réduction des méfaits, il est essentiel de renforcer les capacités fonctionnelles et humaines, le soutien technique, les moyens financiers et l'acceptabilité de ces programmes par la communauté via une démarche de sensibilisation.

Liste des abréviations

CBO : Organisation communautaire, GDT : Groupe de discussion thématique ; VIH : Virus de l'immunodéficience humaine ; EA : Entretien approfondi, KANCO : Kenya AIDS NGO Consortium (Consortium des ONG SIDA au Kenya) ; ONG : Organisation non gouvernementale ; MEWA : Muslim Education and Welfare Association (Association musulmane d'éducation et d'aide sociale) ; SSR : Santé sexuelle et reproductive.

Concernant ce supplément

Cet article a été publié comme partie de l'ouvrage *Reproductive Health*, Volume 16 Supplement 1, 2019: Effective Integration of Sexual Reproductive Health and HIV Prevention, Treatment, and Care Services across sub-Saharan Africa: Where is the evidence for program implementation?
Le supplément a été publié dans le cadre d'une collaboration entre *Reproductive Health* et *BMC Public Health.* L'intégralité du contenu, avec les versions en français, en portugais et en anglais, est disponible en ligne :
https://bmcpublichealth.biomedcentral.com/articles/supplements/volume-19-supplement-1
et
https://reproductive-health-journal.biomedcentral.com/articles/supplements/volume-16-supplement-1

Déclarations

Approbation éthique et accord de participation : Le comité d'éthique de la National Commission for Science Technology and Innovation (Commission nationale kenyane pour les sciences, la technologie et l'innovation) a approuvé la collecte de données primaires dans cette étude (réf. : P/15/8861/4510). Un consentement éclairé écrit a été recueilli auprès de tous les participants.

Accord de publication : L'accord de publication a été obtenu des participants, comme cela est stipulé dans le formulaire de consentement éclairé.

Disponibilité des données et matériels : Les données utilisées dans ce manuscrit ne peuvent pas être rendues publiques en raison des restrictions stipulées dans l'approbation éthique. L'usage de drogues injectables est incriminé et stigmatisé au Kenya. Des demandes raisonnables de données peuvent être adressées à l'auteur correspondant.

Conflits d'intérêts : Les auteurs déclarent ne pas avoir d'intérêts concurrents.

Financement : Les actions menées autour de l'intégration des services SSR et décrites dans ce manuscrit ont été financièrement prises en charge par le fonds d'innovation de l'Alliance internationale contre le VIH/SIDA. Le donateur n'a pas participé à la conception de l'étude et à la collecte, à l'analyse et à l'interprétation des données, ou à la rédaction du manuscrit. Le supplément de la revue est rendu possible grâce au soutien généreux du peuple américain via la United States Agency for International Development (USAID) en partenariat avec le Fonds des Nations unies pour la population (FNUAP) et le Programme commun des Nations Unies sur le VIH/SIDA (ONUSIDA).

Les opinions exprimées dans la présente publication sont celles des auteurs et ne reflètent pas nécessairement les politiques officielles de l'USAID, du FNUAP ou de l'ONUSIDA, la mention des dénominations de ministères ou d'organismes n'implique pas non plus l'aval du gouvernement américain, du FNUAP ou de l'ONUSIDA.

Contributions des auteurs : SA, FJ, FH, AB, TA et GM étaient impliqués dans la conception des actions décrites dans ce document. SA et GM ont participé au développement des outils et du protocole de recherche. GM a analysé les données. SA et GM ont contribué à l'interprétation des résultats et à la rédaction du manuscrit. Tous les auteurs ont lu et approuvé le manuscrit final.

Remerciements : Nous remercions tous les membres de l'équipe de collecte des données, les organisations communautaires, les autorités locales, tous les participants, ainsi que les partenaires chargés de la mise en place du projet, qui ont permis de réaliser cette étude et ces travaux. Le contenu de ce document se trouve sous la responsabilité de ses auteurs et ne reflète pas le point de vue du donateur du projet, des partenaires chargés de sa mise en place ni des institutions respectives auxquelles sont rattachés les auteurs. Les auteurs remercient Taylor & Francis Ltd, http://www.tandfonline.com, de leur avoir permis de réutiliser et/ou de réimprimer le Tableau 1, le Tableau 2 et les extraits de textes associés/sélectionnés, comme stipulé dans les licences numéros : 4492440639418, 4492450569397, 4492450713572 et P122118-06/UWRH. Ces licences incluent les droits d'auteur liés au contenu spécifique de la Society for Menstrual Cycle Research (Société pour la recherche sur le cycle menstruel), www.menstruationresearch.org, que nous avons utilisé avec toutes les autorisations nécessaires.

Références

1. NASCOP: *Kenya AIDS Indicator Survey 2012.* Nairobi: National AIDS and STI Control Programme (NASCOP), Ministry of Health, Kenya; 2014.
2. Petersen Z, Myers B, van Hout MC, Pluddemann A, Parry C: **Availability of HIV prevention and treatment services for people who inject drugs: findings from 21 countries.** *Harm reduction journal* 2013, **10:**13.
3. Mburu G, Limmer M, Holland P: **HIV risk behaviours among women who inject drugs in coastal Kenya: findings from secondary analysis of qualitative data.** *Harm reduction journal* 2019, **16**.
4. NACC: *Kenya AIDS Epidemic Update 2014.* Nairobi: National AIDS Control Council (NACC), Ministry of Health, Government of Kenya; 2014.
5. Tun W, Sheehy M, Broz D, Okal J, Muraguri N, Raymond HF, Musyoki H, Kim AA, Muthui M, Geibel S: **HIV and STI prevalence and injection behaviors among people who inject drugs in Nairobi: results from a 2011 bio-behavioral study using respondent-driven sampling.** *AIDS and behavior* 2015, **19 Suppl 1:**S24-35.
6. Kurth AE, Cleland CM, Des Jarlais DC, Musyoki H, Lizcano JA, Chhun N, Cherutich P: **HIV Prevalence, Estimated Incidence, and Risk Behaviors Among People Who Inject Drugs in Kenya.** *Journal of acquired immune deficiency syndromes (1999)* 2015, **70:**420-427.
7. NASCOP: *Kenya National Guidelines for the Comprehensive Management of the Health Risks and Consequences of Drug Use.* Nairobi: National AIDS and STI Control Programme (NASCOP). Ministry of Health, Government of Kenya; 2013.
8. NACC and NASCOP: *Kenya AIDS Epidemic Update 2011.* Nairobi: National AIDS Control Council (NACC) and National AIDS and STI Control Programme (NASCOP), Ministry of Health, Government of Kenya; 2012.
9. WHO, UNODC, UNAIDS: *Technical Guide for Countries to Set Targets for Universal Access to HIV Prevention, Treatment and Care for Injecting Drug Users.* Geneva, Switzerland: WHO, UNODC and UNAIDS; 2009.
10. Mburu G, Ayon S, Tsai AC, Ndimbii J, Wang B, Strathdee S, Seeley J: **"Who has ever loved a drug addict? It's a lie. They think a 'teja' is as bad person": multiple stigmas faced by women who inject drugs in coastal Kenya.** *Harm reduction journal* 2018, **15:**29.
11. Ayon S, Ndimbii J, Jeneby F, Abdulrahman T, Mlewa O, Wang B, Ragi A, Mburu G: **Barriers and facilitators of access to HIV, harm reduction and sexual and reproductive health services by women who inject drugs: role of community-based outreach and drop-in centers.** *AIDS care* 2017**:**1-8. doi: 10.1080/09540121.09542017.01394965. [Epub ahead of print].
12. NASCOP: *Kenya most at risk population size estimate. Consensus report.* Nairobi: NASCOP, Ministry of Health; 2013.
13. Williams ML, McCurdy SA, Atkinson JS, Kilonzo GP, Leshabari MT, Ross MW: **Differences in HIV risk behaviors by gender in a sample of Tanzanian injection drug users.** *AIDS and behavior* 2007, **11:**137-144.
14. Lambdin BH, Bruce RD, Chang O, Nyandindi C, Sabuni N, Zamudio-Haas S, McCurdy S, Masao F, Ivo Y, Msami A, et al: **Identifying programmatic gaps: inequities in harm reduction service utilization among male and female drug users in Dar es Salaam, Tanzania.** *PloS one* 2013, **8:**e67062.

15. Des Jarlais DC, Feelemyer JP, Modi SN, Arasteh K, Hagan H: **Are females who inject drugs at higher risk for HIV infection than males who inject drugs: an international systematic review of high seroprevalence areas.** *Drug and alcohol dependence* 2012, **124:**95-107.
16. Azim T, Bontell I, Strathdee SA: **Women, drugs and HIV.** *The International journal on drug policy* 2015, **26 Suppl 1:**S16-21.
17. International HIV/AIDS Alliance: *Community Responses to Injecting Drug Use and HIV.* Hove, United Kingdom: International HIV/AIDS Alliance; 2010.
18. Armstrong G: **Commentary on McFall et al. (2017): The need for harm reduction interventions that are effective for women who use drugs.** *Addiction (Abingdon, England)* 2017, **112:**1488-1489.
19. Sherman SG, Kamarulzaman A, Spittal P: **Women and drugs across the globe: a call to action.** *The International journal on drug policy* 2008, **19:**97-98.
20. Greenfield SF, Brooks AJ, Gordon SM, Green CA, Kropp F, McHugh RK, Lincoln M, Hien D, Miele GM: **Substance abuse treatment entry, retention, and outcome in women: a review of the literature.** *Drug and alcohol dependence* 2007, **86:**1-21.
21. Morrison CL, Ruben SM, Beeching NJ: **Female sexual health problems in a drug dependency unit.** *International journal of STD & AIDS* 1995, **6:**201-203.
22. Peters V, Liu KL, Dominguez K, Frederick T, Melville S, Hsu HW, Ortiz I, Rakusan T, Gill B, Thomas P: **Missed opportunities for perinatal HIV prevention among HIV-exposed infants born 1996-2000, pediatric spectrum of HIV disease cohort.** *Pediatrics* 2003, **111:**1186-1191.
23. Tumlinson K, Pence BW, Curtis SL, Marshall SW, Speizer IS: **Quality of Care and Contraceptive Use in Urban Kenya.** *International perspectives on sexual and reproductive health* 2015, **41:**69-79.
24. Mburu G, Ndimbii J, Ayon S, Mlewa O, Mbizvo M, Kihara C, Ragi A: **Contraceptive use among women who inject drugs: Motivators, barriers and unmet needs.** *Women's Reproductive Health* 2018, **5:**99–116.
25. Rhodes T, Closson EF, Paparini S, Guise A, Strathdee S: **Towards "evidence-making intervention" approaches in the social science of implementation science: The making of methadone in East Africa.** *The International journal on drug policy* 2016, **30:**17-26.
26. UNODC: *Community-based treatment and care for drug use and dependence.* Vienna: United Nations Office of Drug and Crime (UNODC); 2014.
27. Coyle SL, Needle RH, Normand J: **Outreach-based HIV prevention for injecting drug users: a review of published outcome data.** *Public health reports (Washington, DC : 1974)* 1998, **113 Suppl 1:**19-30.
28. Kerr T, Hayashi K, Fairbairn N, Kaplan K, Suwannawong P, Zhang R, Wood E: **Expanding the reach of harm reduction in Thailand: experiences with a drug user-run drop-in centre.** *The International journal on drug policy* 2010, **21:**255-258.
29. Ti L, Hayashi K, Hattirat S, Suwannawong P, Kaplan K, Kerr T: **'Drug users stick together': HIV testing in peer-based drop-in centres among people who inject drugs in Thailand.** *Sexual health* 2015, **12:**263-268.
30. Harding C, Ritchie J: **Contraceptive practice of women with opiate addiction in a rural centre.** *The Australian journal of rural health* 2003, **11:**2-6.

31. Black KI, Stephens C, Haber PS, Lintzeris N: **Unplanned pregnancy and contraceptive use in women attending drug treatment services.** *The Australian & New Zealand journal of obstetrics & gynaecology* 2012, **52:**146-150.
32. Palinkas LA, Aarons GA, Horwitz S, Chamberlain P, Hurlburt M, Landsverk J: **Mixed method designs in implementation research.** *Administration and policy in mental health* 2011, **38:**44-53.
33. Creswell J: *Research Design: Qualitative, Quantitative and Mixed Methods Approaches.* Thousand Oaks, CA: Sage; 2003.
34. Esterberg K: *Qualitative methods in social research.* Boston, USA McGraw-Hill; 2002.
35. Bazeley P: *Qualitative data analysis with NVIVO. .* London Sage 2007.
36. Pinkham S, Stoicescu C, Myers B: **Developing effective health interventions for women who inject drugs: key areas and recommendations for program development and policy.** *Advances in preventive medicine* 2012, **2012:**269123.
37. Weber AE, Tyndall MW, Spittal PM, Li K, Coulter S, O'Shaughnessy MV, Schechter MT: **High pregnancy rates and reproductive health indicators among female injection-drug users in Vancouver, Canada.** *The European journal of contraception & reproductive health care : the official journal of the European Society of Contraception* 2003, **8:**52-58.
38. Ralph N, Spigner C: **Contraceptive practices among female heroin addicts.** *American journal of public health* 1986, **76:**1016-1017.
39. Terplan M, Hand DJ, Hutchinson M, Salisbury-Afshar E, Heil SH: **Contraceptive use and method choice among women with opioid and other substance use disorders: A systematic review.** *Preventive medicine* 2015, **80:**23-31.
40. Than LC, Honein MA, Watkins ML, Yoon PW, Daniel KL, Correa A: **Intent to become pregnant as a predictor of exposures during pregnancy: is there a relation?** *The Journal of reproductive medicine* 2005, **50:**389-396.
41. Olsen A, Banwell C, Madden A: **Contraception, punishment and women who use drugs.** *BMC women's health* 2014, **14:**5.
42. Shaw R, Samaha H: *Building capacity for health system strengthening: a strategy that works.* Washington DC: World Bank Institute; 2009.
43. Ekirapa A, Mgomella GS, Kyobutungi C: **Civil society organizations: capacity to address the needs of the urban poor in Nairobi.** *Journal of public health policy* 2012, **33:**404-422.
44. Ippoliti NB, Nanda G, Wilcher R: **Meeting the Reproductive Health Needs of Female Key Populations Affected by HIV in Low- and Middle-Income Countries: A Review of the Evidence.** *Studies in family planning* 2017, **48:**121-151.
45. Armstrong KA, Kenen R, Samost L: **Barriers to family planning services among patients in drug treatment programs.** *Family planning perspectives* 1991, **23:**264-266, 270-261.
46. Islam MM, Topp L, Conigrave KM, van Beek I, Maher L, White A, Rodgers C, Day CA: **The reliability of sensitive information provided by injecting drug users in a clinical setting: clinician-administered versus audio computer-assisted self-interviewing (ACASI).** *AIDS care* 2012, **24:**1496-1503.

Tableaux

Tableau 1. SEQ Table * ARABIC 1. Comportements en matière de fertilité et de contraception à la période de référence.

Caractéristique	**EA**	**GDT**	**Total**	**%**
Nombre d'enfants				
(moyenne)	1,4	1,8	1,6	-
**Méthode de**				
**contraception actuelle**				
Préservatifs	5	1	6	13 %
Calendrier	1	0	1	2 %
Implant	2	0	2	4 %
Plantes	1	0	1	2 %
Aucune	13	18	31	69 %
Injections	1	2	3	7 %
Inconnue	1	0	1	2 %

Tableau 2. Services étendus fournis aux femmes faisant usage de drogues injectables sur les sites de l'étude.

Domaine concerné par les services	Interventions effectuées et services proposés pendant les actions de proximité	Interventions effectuées et services proposés dans les centres d'accueil	Orientations vers des services sanitaires et sociaux privés et gouvernementaux
Prévention et traitement du VIH et des co-infections.	Préservatifs, tests de dépistage du VIH, information, communication et éducation sur le VIH et les infections sexuellement transmissibles.	Tests de dépistage du VIH et conseils	Orientation vers un autre établissement pour confirmation et traitement du VIH, et dépistage de l'hépatite C et de la tuberculose.
Réduction des méfaits.	Aiguilles et seringues propres, tampons imbibés d'alcool, coton.	Conseils sur l'addiction et premiers secours en cas de violence et d'overdose.	Orientations vers un traitement médicalement assisté à base de méthadone.
Services de santé sexuelle et reproductive.	Informations sur la planification familiale, conseils entre pairs, protections hygiéniques/tampons et pilules contraceptives.	Éducation sur les soins prénataux et contraceptifs réversibles de courte durée.	Orientation vers une contraception réversible à action prolongée, des soins prénataux et un dépistage du cancer du col de l'utérus.
Services personnels, sociaux et de garde d'enfants.	Transport vers les établissements de santé et fourniture de produits de soins personnels.	Soins personnels (gel douche, brosse à dents/dentifrice, crème), hébergement de courte durée et couches pour les enfants.	Orientation vers des services d'assistance juridique et d'aide en cas de violence sexuelle.
