# Supplementary material for: Developing integrated community-based HIV prevention, harm reduction, and sexual and reproductive health services for women who inject drugs
Source: Reprod Health. 2019 May 29;16(Suppl 1):59. doi: 10.1186/s12978-019-0711-z (PMC6538559; doi:10.1186/s12978-019-0711-z)
Supplement: Supplementary file 2 — Translation of the abstract of this article into Portuguese. (PDF 96.7 kb) [file 12978_2019_711_MOESM2_ESM.pdf]

Reproductive Health 2019, 16 (Suppl 1) [translated from the original publication]

Desenvolvimento de serviços comunitários integrados de prevenção e redução dos danos do VIH e de saúde sexual e reprodutiva para mulheres consumidoras de drogas intravenosas

Sylvia Ayon[1], Fatma Jeneby[2], Faizah Hamid[3], Abdalla Badhrus[2], Taib Abdulrahman[3], Gitau Mburu[4*]

Afiliações:
Kenya AIDS NGO Consortium, Nairobi, Kenya.
Muslim Education and Welfare Association, Mombasa, Kenya.
REACHOUT Trust, Mombasa, Kenya.
Division of Health Research, University of Lancaster, Lancaster, United Kingdom.

Endereços de e-mail:
SA: sayon@kanco.org
FJ: fjeneby@yahoo.com
BH: bintyhamid@gmail.com
AB: abdallabadhrus@yahoo.com
TA: twayeebahmed@gmail.com
GM: g.mburu@lancs.ac.uk
*Autor correspondente: Gitau Mburu, Division of Health Research, University of Lancaster, Lancaster, LA1 4YW, United Kingdom.
Email: g.mburu@lancs.ac.uk

Resumo

Introdução: As necessidades ao nível de saúde sexual e reprodutiva (SSR) das mulheres consumidoras de drogas intravenosas estão a ser negligenciadas, apesar de serem uma população prioritária para os programas de prevenção e redução dos danos do VIH. Adicionalmente, os modelos para fornecer os serviços integrados de SSR, VIH e redução dos dados para mulheres consumidoras de drogas intravenosas são raros. Este artigo aborda o desenvolvimento de serviços comunitários de proximidade que integraram o planeamento familiar e outras intervenções de SSR com serviços de VIH e redução dos danos para esta população da região costeira do Quénia.

Métodos: Foi realizada uma avaliação qualitativa das necessidades iniciais com mulheres consumidoras de drogas intravenosas e partes interessadas na redução dos danos, utilizando uma combinação de inquéritos exaustivos, debates em grupo e metodologia de investigação-ação. Os dados qualitativos dos participantes foram sujeitos a uma análise temática através do software Nvivo. Tendo por base a avaliação das necessidades iniciais, a integração de SSR em serviços existentes de VIH e de redução dos danos foi implementada. Após dois anos de implementação, foi efetuada uma avaliação ao programa utilizando uma combinação de entrevistas qualitativas e revisão dos registos quantitativos da prestação do serviço, bem como outros documentos do programa. O processo, os impactos e os desafios da integração da SSR em programas comunitários de prevenção e redução dos danos do VIH

foram identificados.

Resultados: Este artigo destaca: 1) a escassa utilização dos serviços de planeamento familiar entre mulheres consumidoras de drogas intravenosas; 2) uma melhor utilização e elevada aceitação da prestação de serviços de SSR com base na proximidade, incluindo contraceção nesta população; 3) a importância de formação, fortalecimento das capacidades, apoio técnico e cobertura financeira para permitir que as organizações comunitárias integrem serviços de SSR em serviços de prevenção e redução dos danos do VIH; e 4) o valor da participação dos beneficiários, defesa e colaboração com outros parceiros na elaboração, no planeamento e na implementação de intervenções de SSR para mulheres consumidoras de drogas intravenosas.

Conclusões: As mulheres consumidoras de drogas intravenosas neste estudo apresentaram uma baixa utilização dos serviços de planeamento familiar e de outros serviços de SSR, os quais podem ser melhorados através da integração de intervenções de contraceção e outras intervenções de SSR em programas existentes para prevenção e redução dos danos do VIH. Esta integração é viável de forma programática e aceitável para mulheres consumidoras de drogas intravenosas. Para obter uma integração bem-sucedida, é essencial ter uma participação dos beneficiários baseada em direitos, juntamente com um fortalecimento das capacidades técnicas e financeiras sustentáveis a nível comunitário.

Palavras-chave: VIH, saúde reprodutiva, contraceção, redução de danos, heroína, integração, género, Quénia.

Sobre este suplemento

Este resumo foi publicado como parte da revista científica *Reproductive Health*, Volume 16, Suplemento 1, 2019: Integração Eficaz dos Serviços de Saúde Sexual e Reprodutiva e de Prevenção, Cuidados e Tratamento do VIH na África Subsariana: Onde estão as provas da implementação do programa?
O suplemento foi publicado como uma colaboração entre as revistas científicas *Reproductive Health* e *BMC Public Health.* O conteúdo integral, incluindo as versões em francês, português e inglês, estão disponíveis online:
https://bmcpublichealth.biomedcentral.com/articles/supplements/volume-19-supplement-1
e
https://reproductive-health-journal.biomedcentral.com/articles/supplements/volume-16-supplement-1
